# Relative Contribution of Cellular Complement Inhibitors CD59, CD46, and CD55 to Parainfluenza Virus 5 Inhibition of Complement-Mediated Neutralization

**DOI:** 10.3390/v10050219

**Published:** 2018-04-25

**Authors:** Yujia Li, Griffith D. Parks

**Affiliations:** Burnett School of Biomedical Sciences, College of Medicine, University of Central Florida, Orlando, FL 32827, USA; Mli@jcvi.org

**Keywords:** complement, cell surface inhibitors, virus neutralization

## Abstract

The complement system is a part of the innate immune system that viruses need to face during infections. Many viruses incorporate cellular regulators of complement activation (RCA) to block complement pathways and our prior work has shown that Parainfluenza virus 5 (PIV5) incorporates CD55 and CD46 to delay complement-mediated neutralization. In this paper, we tested the role of a third individual RCA inhibitor CD59 in PIV5 interactions with complement pathways. Using a cell line engineered to express CD59, we show that small levels of functional CD59 are associated with progeny PIV5, which is capable of blocking assembly of the C5b-C9 membrane attack complex (MAC). PIV5 containing CD59 (PIV5-CD59) showed increased resistance to complement-mediated neutralization in vitro comparing to PIV5 lacking regulators. Infection of A549 cells with PIV5 and RSV upregulated CD59 expression. TGF-beta treatment of PIV5-infected cells also increased cell surface CD59 expression and progeny virions were more resistant to complement-mediated neutralization. A comparison of individual viruses containing only CD55, CD46, or CD59 showed a potency of inhibiting complement-mediated neutralization, which followed a pattern of CD55 > CD46 > CD59.

## 1. Introduction

The complement system is a major component of innate immunity that most animal viruses must face during natural infections. Complement consists of both soluble and cell membrane-associated proteins that integrate host defenses to viral infections. This includes a variety of mechanisms such as recognition and neutralization of viruses, recruitment and stimulation of leukocytes, opsonization of viruses by immune cells, and activation of B and T cell immunities [1,2,3]. Viruses have also evolved to block complement pathways and this may contribute to viral pathogenesis and disease [4,5,6,7]. Studies have shown that a better understanding of virus-complement interactions will be necessary for designing more effective vaccines and therapeutic vectors [8,9,10]. The overall goal of this work was to determine the role of an important host regulator of complement CD59 in limiting neutralization of Parainfluenza virus 5 (PIV5).

The complement system is activated through one or more main pathways including the classical pathway, lectin pathway, and alternative pathways [11,12]. Once activated, these three pathways converge on a central complement component C3, which is cleaved into C3a and C3b. C3b can bind covalently to viral components to aid in opsonization and phagocytosis. In addition, C3b can associate with other complement components such as cleavage products from Factor B to form the C3 convertase. This leads to amplification of the initially deposited C3b by further cleavage of C3 molecules to form a feedback loop. C3b can associate with downstream complementary components to drive further cleavage reactions and with the membrane attack complex (MAC), which is capable of lysing virus particles or infected cells [2,7,13]. In this case, C5 is cleaved to C5a and C5b with the latter of which associates with C6, C7, and C8 to form a pre-MAC complex. Ultimately, the complex drives polymerization of C9 to associate with the membrane and form a pore that lyses cells.

Activation of the complement system is tightly regulated to avoid damage to normal cells and healthy tissues [14,15]. This regulation involves the actions of soluble and cell-associated proteins called regulators of complement activation (RCA) [16]. Three important RCAs include CD46, CD55, and CD59. Membrane-bound CD46 acts as a co-factor along with factor I protease to cleave C3b in the C3 convertase or C4b in the C4 convertase into inactive products. CD55 also targets the C3 and C4 convertases, but acts to dissociate these complexes for inactivation. The membrane-bound RCA protein CD59 is the major controller of MAC formation. CD59 attaches to the host cell through the glycosyl phosphatidyl inositol (GPI) anchor [17,18]. CD59 inhibits MAC formation through two major mechanisms such as by binding tightly to the C5b-8 complex to inhibit the incorporation of multiple C9 molecules and by inhibiting C9 polymerization [19,20,21].

Enveloped viruses have evolved specific mechanisms to evade the complement system. Many of these are based on exploiting these host cell RCAs. First, viruses can encode proteins that mimic the structure and function of host RCAs. For example, vaccinia virus encodes a secreted soluble protein called the complement control protein to induce the dissociation of C3 convertase [22]. Second, viruses can recruit soluble RCAs to limit complement activation. For instance, the West Nile virus encodes cell-surface-associated and soluble forms of NS1 that recruits the complement regulatory protein factor H. This results in limited complement activation and deposition of C3 and MAC on infected cell surfaces [23]. Next, viruses can recruit cell membrane-associate RCAs into their virions during the budding process at the host plasma membrane [7,24]. This includes studies with the influenza virus, the vaccinia virus, and human immunodeficiency virus type 1 (HIV 1), which have been shown to incorporate CD55, CD59, and/or CD46 into progeny virions [25,26,27,28].

Parainfluenza virus 5 (PIV5) activates the alternative pathway of complement and this activation can contribute to the efficiency of neutralization by human serum [29,30]. We have previously shown that PIV5 incorporates CD46 and CD55 into progeny virions. Both of these virion-associated RCAs can contribute to complement inhibition with CD55 being much more potent than CD46 for limiting complement-mediated neutralization in vitro [31,32]. Further studies have shown that PIV5 infection upregulates CD55 expression. In addition, PIV5 derived from cells with higher cell surface CD55 was more resistant to neutralization by normal human serum [33].

In this paper, we have extended our studies on PIV5-association with host cell RCAs to test the role of CD59 in limiting complement-mediated PIV5 neutralization. Using cells which were engineered to express CD59 as the only RCA, we show that PIV5 incorporates small amounts of CD59 into progeny virions and that virion-associated CD59 is functional in inhibiting complement pathways to limit neutralization. A comparison of individual viruses containing only CD55, CD46, or CD59 showed a potency of inhibiting complement-mediated neutralization, which followed a pattern of CD55 > CD46 > CD59. Infection with PIV5 as well as other RNA viruses results in increased levels of CD59 on the cell surface. PIV5 derived from cells with higher cell surface CD59 was more resistant to neutralization. We propose a model for the role of virus induction of CD59 in defining virus growth and tissue tropism.

## 2. Materials and Methods

### 2.1. Cells and Viruses

CV-1, MDBK, Vero, A549, and HeLa cells were grown in Dulbecco modified eagle medium (DMEM) supplemented with 10% heat inactivated (HI) fetal bovine serum (FBS). To generate CHO cells stably expressing CD59 (CHO-CD59), total RNA was purified from 10^5^ A549 cells using TRIzol (Invitrogen, Carlsbad, CA, USA) and 1 μg of RNA was used to generate a cDNA by reverse transcription-PCR. Products were digested with EcoRI and XhoI and cloned into the corresponding sites of pcDNA3.1. CHO cells were transfected with the plasmid encoding CD59 followed by single cell sorting using an antibody to CD59. All CHO cells were maintained in DMEM supplemented with 4.5 g/L glucose, 10 mM HEPES pH 7.2, 4 mM l-glutamine, 1% nonessential amino acid, and 6% HI FBS. PIV5 was grown in MDBK cells. The Wake Forest strain of PIV5 [34], PIV5 leader mutant (PIV5 Le mutant) [35], Mumps virus (MuV), Zika virus PRV59 strain (ZIKV PRV), and vesicular stomatitis virus (VSV) were grown in Vero cells. Human parainfluenza virus type 2 (PIV2) and Respiratory Syncytial Virus (RSV) were grown in CV-1 cells.

### 2.2. Ultracentrifugation and Western Blotting

PIV5 that was grown in CHO-CD59 cells (PIV5-CD59) was pelleted by ultracentrifugation (25,000 RPM, 4 h, SW28 rotor) and then further purified on 20% to 60% sucrose gradients, which was detailed previously [32]. Fractions collected from the bottom of the tube were analyzed by Western blotting with rabbit serum specific for the PIV5 P protein or with rabbit anti-human CD59 antibody (Cell Signaling Technology, Danvers, MA, USA). In the case of CHO-CD59 cells, lysates were also probed with mouse anti-human actin to normalize loading amounts. To quantitate CD59 levels, dilutions of purified virus were analyzed by Western blotting along with known amounts of recombinant human CD59 protein (EMC Biochemical, Hopkinton, MA, USA). Blots were visualized by horseradish peroxidase-conjugated antibodies and chemiluminescent substrate (Thermo Scientific, Waltham, MA, USA).

### 2.3. Neutralization Assay

Normal human serum (NHS) was collected from healthy donors, processed, and divided into small aliquots before freezing at −80 °C, which was described previously [36]. Sera were heat inactivated (HI) by heating to 56 °C for 30 min to abolish the complement. C5-depleted and C8-depleted serum were purchased from Complement Technologies (Tyler, TX, USA). Time or dose-dependent neutralization assays were carried out as described previously [36]. One hundred plaque forming units (PFU) of PIV5 was incubated at 37 °C with various dilutions of NHS for 1 h or alternatively with a constant dilution of NHS for various times, which is described in the figure legends. Remaining infectivity was determined by using a plaque assay on CV-1 cells. The reported results were the averages of three independent reactions. Statistical analysis was performed to calculate p values using a student’s *t*-test.

### 2.4. Flow Cytometric Analysis

Cell surface expression of C3, C5b-9, and CD59 was analyzed as described earlier [33]. Virus infected or mock infected cells were trypsinized, suspended in DMEM, supplemented with 10% HI FBS, and then washed once with PBS. Cell surface CD59 was detected by using PE-conjugated anti-human CD59 antibody (BioLegend, San Diego, CA, USA) at a 1:100 dilution. Flow cytometric analysis was performed using the CytoFLEX Flow Cytometer (Beckman Coulter, Brea, CA, USA) recording at least 10,000 independent events. Results were analyzed using CytExpert software (Beckman Coulter, Indianapolis, IN, USA).

### 2.5. Reverse Transcription and Real Time PCR

Total RNA was purified from 10^5^ cells using TRIzol (Invitrogen, Carlsbad, CA, USA) and 1 μg of RNA was used to generate cDNA using the TaqMan^®^ Reverse Transcription Reagents (Applied Biosystems, Foster City, CA, USA) as described by the manufacturer. Quantitative real-time PCR was carried out by using the Fast SYBR^®^ FAST Green Master Mix (Applied Biosystems) and Bio-Red CFX Connect Real-Time. Primers used include: CD59 forward 5′-AAGAAGGACCTGTGTAACTT-3′, CD59 reverse 5′-GAGTCACCAGCAGAAGAA-3′; β-actin forward 5′-GATCATTGCTCCTCCTGAGC-3′, and β-actin reverse 5′-ACTCCTGCTTGCTGATCCAC-3′. Relative CD59 replication was analyzed using the web based service (RT^2^ Profiler PCR analysis, SABiosciences, Hilden, Germany), which was described previously [37].

### 2.6. C9 Polymerization Assay

Polymerization of C9 was assayed by native gel electrophoresis as described previously [38,39]. Purified PIV5 (4 μg) or PIV5-CD59 (1 to 4 μg) was pre-incubated with 2 μg of purified C9 (Complement Technologies, Tyler, TX, USA) at 37 °C for 40 min. Polymerization was induced by adding ZnCl_2_ to a final concentration of 50 μM and incubation for a further 2 h at 37 °C. The samples were separated in precast 4% to 20% gradient polyacrylamide gels (Bio-Rad, Hercules, CA, USA) under the native condition. C9 polymerization was visualized by silver staining or Western blotting using the mouse anti-C5b-9 antibody (Quidel, San Diego, CA, USA). Relative band densities of C9 monomer were measured by ImageJ (National Institutes of Health, Bethesda, MD, USA).

### 2.7. Complement Deposition on Infected Cells

CHO and CHO-CD59 cells were mock infected or infected with PIV5-GFP at an moi of 10. At 2 dpi, cells were treated with serum-free DMEM or with HI NHS or NHS at a dilution of 1:10 for 1 h. C3 and C5b-9 deposition on the cell surface was detected by flow cytometry using mouse anti-C5b-9 antibody (Quidel, San Diego, CA, USA). In a parallel experiment, CHO and CHO-CD59 cells were infected with PIV5 at an moi of 10. At 2 dpi, cells were treated with NHS at a dilution of 1:10 for 1 h or with NHS at dilutions of 1:10 and 1:4 for 4 h. Cells were then stained with PE-conjugated Annexin V before analysis with flow cytometry.

## 3. Results

### 3.1. CD59 Is Associated with PIV5 Virions

**Figure 1:**
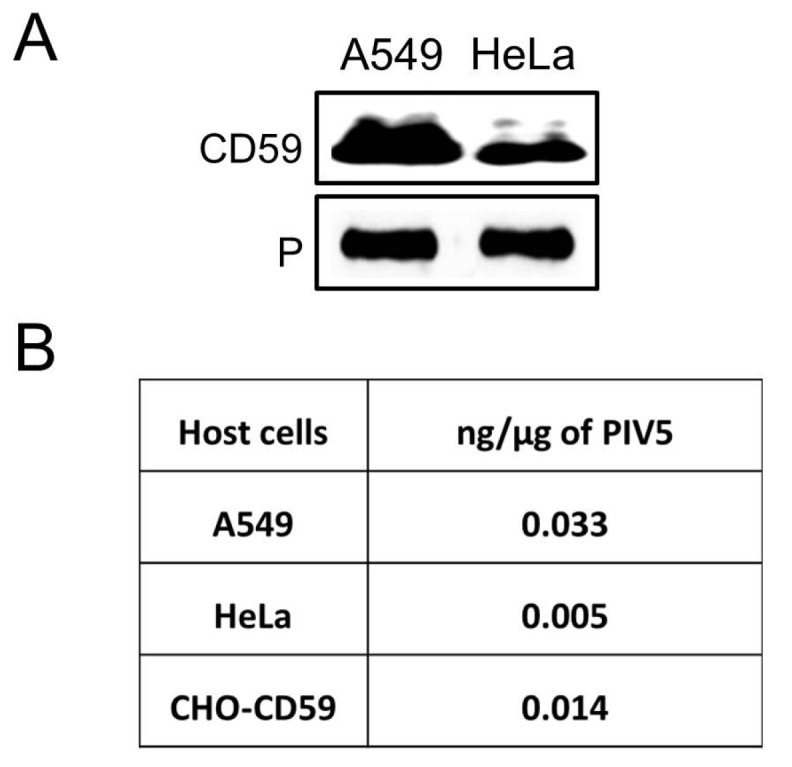
CD59 is associated with PIV5 virions. (**A**) PIV5 derived from the indicated cell lines was purified by gradient ultracentrifugation and analyzed by Western blotting for the presence of CD59. Levels of viral P were analyzed as a loading control. (**B**) PIV5 derived from the indicated cells was analyzed by quantitative Western blotting against known amounts of purified CD59. Relative amounts of CD59 are expressed as ng/μg of purified virus. Results are the average of two independent experiments.

We tested the hypothesis that PIV5 would associate with CD59. A549 and HeLa cells were infected at high moi with PIV5 and the progeny virus was purified by sucrose gradient ultracentrifugation before Western blotting for the presence of CD59. As shown in Figure 1A, CD59 was detected in virions derived from both A549 and HeLa cells. Quantitative Western blotting indicated that on average A549- and a Hela-derived virus contained 0.033 and 0.005 ng of CD59 per μg of virus (see Figure 1B).

It is known that tissues and cell types can vary widely in CD59 expression [17,18] and that many cancer cell types express increased levels of CD59 [40,41,42,43,44]. However, studies to address roles of individual RCAs using viruses from these cell types are complicated since they express multiple RCAs. Therefore, for our study, we utilized Chinese Hamster Ovary (CHO) cells since they do not express any human RCAs.

**Figure 2:**
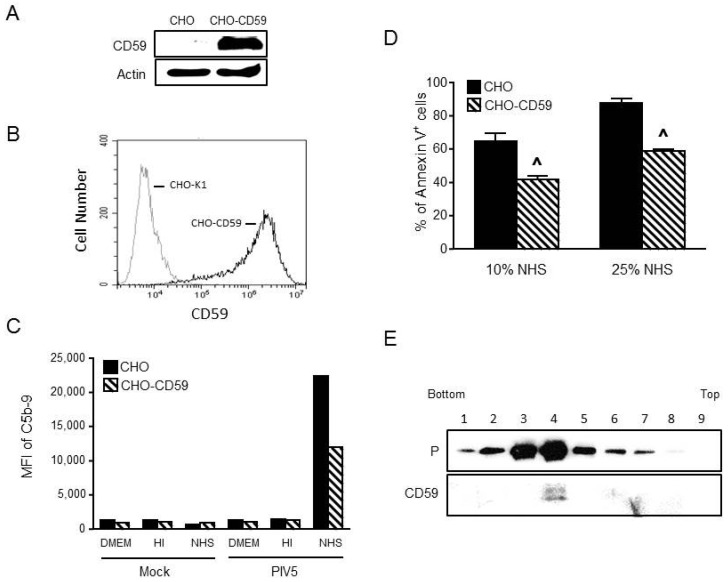
CHO cells expressing CD59 are functional in limiting MAC formation. (**A**,**B**) Parental CHO or CHO cells stably expressing CD59 were analyzed for CD59 expression by Western blotting (panel (**A**)) or by flow cytometry (panel (**B**)). In panel (**A**), levels of actin were analyzed as a loading control. (**C**) CHO and CHO-CD59 cells were mock infected or infected with PIV5 at an moi of 10. At 2 dpi, cells were treated with DMEM as a negative control and HI NHS or NHS at a dilution of 1:10 for 1 h. Cells were analyzed for levels of cell surface C5b-9 by flow cytometry. Results are representative of two independent experiments. (**D**) CHO or CHO-CD59 cells were infected with PIV5 at an moi of 10. At 2 dpi, cells were treated with 10% or 25% NHS for 4 h followed by Annexin V staining and quantitation by flow cytometry. Data are from three independent reactions and error bars represent standard deviation. ˄ *p* < 0.01 when comparing PIV5 infected CHO-CD59 cells to PIV5 infected CHO control cells. (**E**) PIV5 derived from CHO-CD59 cells was sedimented through a 20% to 60% sucrose gradient. Fractions were collected and analyzed by Western blotting for viral P protein or for CD59.

To produce cells which express CD59 as the only complement factor, CHO cells were transfected with a plasmid expressing human CD59 gene and cells stably expressing CD59 were selected by flow cytometric cell sorting. As shown in Figure 2A,B, CD59 expression was confirmed in CHO-CD59 cells by Western blotting and was shown to be expressed at the cell surface by flow cytometry. The parental CHO cells showed no detectable level of CD59.

To test the function of CHO-expressed CD59, parental CHO cells and CHO-CD59 cells were mock infected or infected with PIV5 at an moi of 10. At 2 dpi, cells were treated with DMEM alone and HI NHS or NHS at a dilution of 1:10 for 1 h. MAC deposition on the cell surface was then examined by flow cytometry using an antibody to C5b-9. Figure 2C shows an example of a typical result. There was a higher level of MAC deposition observed for PIV5-infected CHO cells that were treated with NHS compared to cells treated with DMEM or HI NHS. This is consistent with the ability of PIV5 to remove sialic acid from the surface of infected cells [30], which is a strong signal for complement deposition. In the case of PIV5 infected CHO-CD59 cells, there was a decrease in MAC formation compared to infected parental CHO cells.

To further confirm the function of CD59 in limiting MAC-induced cytopathic effects on PIV5-infected cells, CHO or CHO-CD59 cells were infected with PIV5 and then treated with 10% or 25% NHS for 4 h before staining with Annexin V. As shown in Figure 2D, PIV5-infected CHO cells showed ~64% and 90% Annexin V positive staining with 10% and 25% NHS, respectively. In contrast, PIV5 infected CHO-CD59 cells showed a significantly lower percentage of Annexin V positive cells with ~40% and 55% Annexin V positive staining after treatment with 10% and 25% NHS, respectively. Together, these data show that complement is activated on the surface of PIV5-infected CHO cells, which results in MAC formation and early cytopathic effects and that the CD59 expressed in CHO-CD59 cells is functional in limiting these complementary activities.

To generate a virus that contains CD59 as the only complement regulator, PIV5 was grown in CHO-CD59 cells and was purified by ultracentrifugation through 20% to 60% sucrose gradients. Fractions were collected and analyzed by Western blotting for co-sedimentation of CD59 with virions. Rabbit anti-PIV5 P serum was used to localize PIV5 virions and goat anti-human CD59 antibody was used to detect the presence of CD59. As shown in Figure 2E, PIV5 virions and CD59 were co-sedimented in the gradient with a peak in fraction 4. Quantitative Western blotting showed that the CD59 level in PIV5 from CHO-CD59 cells was 0.014 ng/μg virus, which is higher than the Hela cell-derived virus but lower than the value in the A549-derived virus (see Figure 1B).

### 3.2. CD59 Contained in PIV5-CD59 Inhibits C9 Polymerization

**Figure 3:**
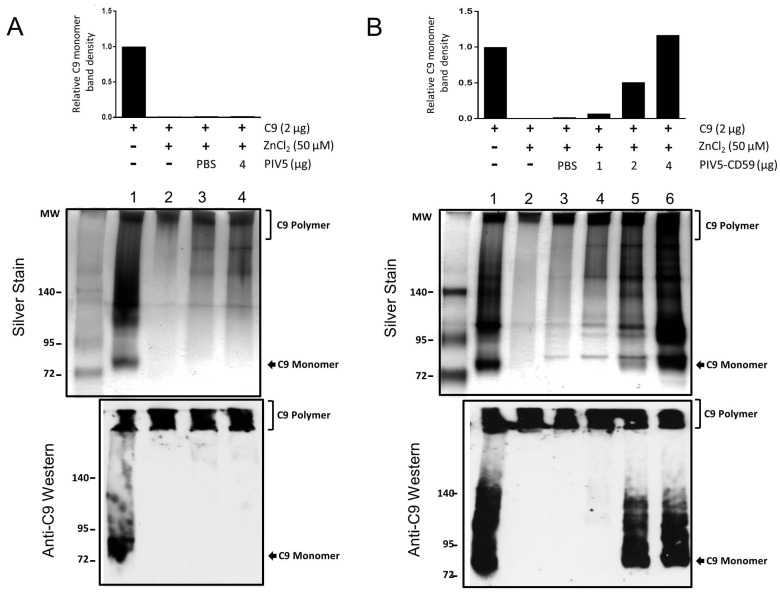
Effect of PIV5-associated CD59 on C9 polymerization in vitro. Purified C9 was incubated alone or with the indicated amounts of PIV5 (**A**) or PIV5-CD59 (**B**) at 37 °C for 40 min. Polymerization of C9 was initiated by adding ZnCl_2_ to 50 μM and by further incubation for 2 h at 37 °C. Samples were separated through 4% to 20% gradient polyacrylamide gels under non-reducing conditions and C9 polymerization was visualized by silver staining (upper panel) or by Western blotting using the anti-C5b-9 antibody (lower panel). The C9 monomer band intensity was measured by Image J, which is shown in the upper panel. These results are an example of three independent experiments.

CD59 can prevent MAC formation by inhibiting C9 polymerization [19,20,21]. An established assay for in vitro C9 polymerization [38] was carried out to determine if the CD59 contained within PIV5-CD59 was functional. The C9 monomer was incubated with PBS as a control or with gradient-purified PIV5 or PIV5-CD59 for 40 min. C9 polymerization was then induced with 50 μM ZnCl_2_ for 2 h and then samples were analyzed by electrophoresis through 4% to 20% gradient polyacrylamide gels under non-reducing conditions. C9 polymerization was visualized by silver staining or by Western blotting for the presence of the C9 monomer. As shown in Figure 3A, C9 existed as a monomer as well as higher molecular weight oligomers (lane 1) and was polymerized to exclusively high molecular weight species by ZnCl_2_ treatment (lane 2). When incubated with PBS or purified PIV5, there was no effect on polymerization (lane 3 and lane 4).

**Figure 4:**
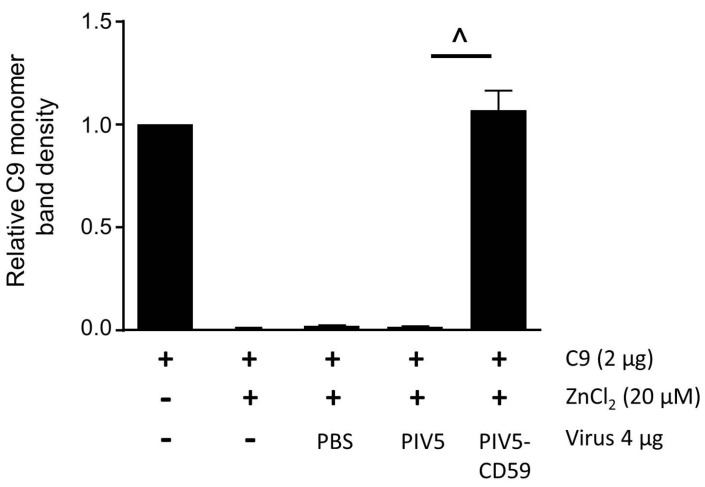
PIV5-CD59 inhibits C9 polymerization in vitro. PIV5-CD59 was assayed for the ability to inhibit polymerization of purified C9, which was described in the legend to Figure 3. The data are from three independent reactions and error bars represent standard deviation. ˄ *p* < 0.01 when comparing results with PIV5 to PIV5-CD59.

By contrast, when C9 was incubated with purified PIV5-CD59 prior to ZnCl_2_ treatment, there was a dose-dependent shift in C9 down to monomeric or multimers (see Figure 3B, lanes 4–6). Increased C9 monomer band density was detected by Western blotting using 4 μg of PIV5-CD59. When the results from three independent experiments were combined, there was a clear inhibition of C9 polymerization when incubated with purified PIV5-CD59 (see Figure 4). Taken together, these data indicate that CD59 associated with PIV5 virions was functional in inhibiting C9 polymerization.

### 3.3. PIV5 Derived from CD59-Expressing Cells Has Increased Resistance to Complement-Mediated Neutralization In Vitro

**Figure 5:**
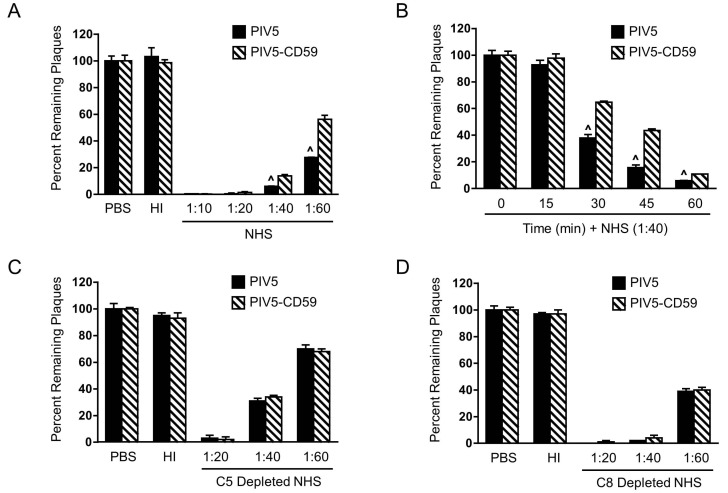
Effect of NHS on neutralization of CD59-associated PIV5. One hundred PFU of PIV5 derived from control CHO cells (PIV5) or CHO-59 cells (PIV5-CD59) was incubated with the indicated dilutions of NHS for 1 h (**A**) or with NHS at a dilution of 1:40 for the indicated times (**B**). The remaining infectivity was determined by using the plaque assay. (**C**,**D**) Serum depleted of C5 (**C**) or depleted of C8 (**D**) was tested as described for panel A for neutralizing PIV5 or PIV5-CD59. As a control, the virus was incubated with PBS alone for 1 h. Data are from three independent reactions and error bars represent standard deviation. ˄ *p* < 0.01 when comparing PIV5 derived from CHO-CD59 cells to PIV5 derived from CHO control cells.

To determine the effect of PIV5-associated CD59 on complement-mediated neutralization, PIV5 was grown in CHO-CD59 and control CHO cells. Stocks were named as PIV5-CD59 and PIV5, respectively. One hundred PFU of virus was incubated with dilutions of NHS for 1 h and the remaining infectivity was determined by using the plaque assay. As shown in Figure 5A, PIV5-CD59 (hatched bars) and PIV5 (black bars) were completely neutralized with 1:10 and 1:20 dilutions of NHS while HI NHS had no effect on infectivity. At 1:40 and 1:60 dilutions of NHS, PIV5-CD59 was more resistant than PIV5 to complement-mediated neutralization. In time-course experiments (see Figure 5B) performed with 1:40 dilution of NHS, PIV5-CD59 was more resistant to neutralization than PIV5 alone (30 min time-point), which suggests that PIV5-associated CD59 has a much greater impact on delaying neutralization than PIV5 with no regulators.

**Figure 6:**
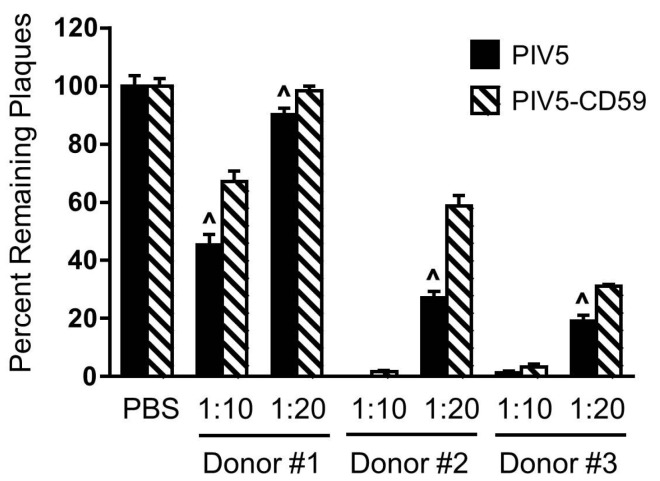
Effect of CD59 on NHS neutralization of PIV5. One hundred PFU of PIV5 derived from control CHO cells (PIV5) or CHO-CD59 cells (PIV5-CD59) was incubated with NHS from the indicated donors at a dilution of 1:10 or 1:20 for 1 h. The remaining infectivity was determined by using the plaque assay. The data are from three independent reactions and error bars represent standard deviation. ˄ *p* < 0.01 when comparing a virus derived from CHO-CD59 cells to a virus derived from CHO control cells.

Given that CD59 functions at the level of C5 and C8, it would be expected that differences in neutralization between PIV5 and PIV5-CD59 would not be seen with C5-depleted or C8-depleted serum. As shown in Figure 5C,D, both viruses were neutralized at 1:20, 1:40, and 1:60 dilutions of NHS to varying degrees. In addition, there was no significant difference between PIV5 and PIV5-CD59 in sensitivity to C5-depleted or C8-depleted serum. The serum used in Figure 5D was different from that in Figure 5A and cannot be directly compared. Using multiple donor serum, PIV5-CD59 was found to be more resistant to complement-mediated neutralization compared to PIV5 that lacked regulators (see Figure 6). However, the level of neutralization differed somewhat between donors, which was previously reported [36]. Together, these data indicate that PIV5 containing CD59 is more resistant to complement-mediated neutralization and this resistance is dependent on C5 and C8.

### 3.4. Relative Contribution of CD59, CD46, and CD55 to PIV5 Complement-Mediated Neutralization

**Figure 7:**
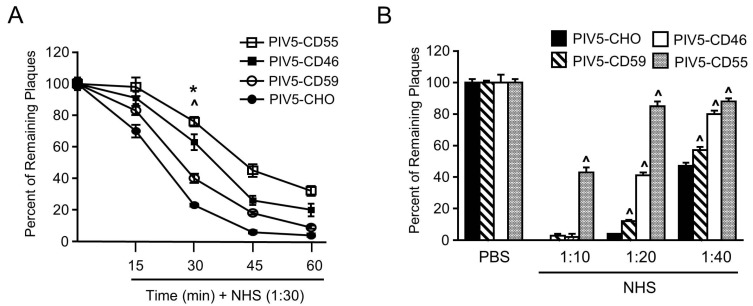
Relative contribution of CD55, CD46, and CD59 to resistance of PIV5 to complement-mediated neutralization. (**A**) One hundred PFU of PIV5 derived from the indicated CHO cell line was incubated with a 1:30 dilution of NHS for the indicated time points. Remaining infectivity was determined by using the plaque assay. The data are from three independent reactions and error bars represent standard deviation. ˄ *p* < 0.01 when comparing CHO-CD46, CHO-CD55, or CHO-CD59 cell-derived PIV5 to CHO control cell-derived PIV5 at 30 min time point. * *p* < 0.01 when comparing CHO-CD46 or CHO-CD55 cell derived PIV5 to CHO-CD59 cell derived PIV5 at a 30 min time point. (**B**) One hundred PFU of PIV5 derived from indicated cell lines was incubated with the indicated dilutions of NHS for 30 min. Remaining infectivity was determined by plaque assay. ˄ *p* < 0.01 when comparing PIV5 from CHO-CD55, CHO-CD46, or CHO-CD59 to PIV5 from CHO control cells.

To directly compare the effect of virion-associated RCAs CD55, CD46, and CD59 on complement-mediated PIV5 neutralization, time courses of neutralization were performed using a virus derived from previously described CHO cells engineered to express CD46, CD55, and CD59 [31,32]. As shown in Figure 7A, incubation of a 1:30 dilution of NHS with 100 PFU of PIV5 from CHO cells resulted in very rapid neutralization with almost no infectivity remaining by 45 min (bottom line). By contrast, PIV5-CD59, PIV5-CD46, and PIV5-CD55 showed an overall increase in resistance to neutralization when compared with PIV5, which is most evident at 30 min. At this time point, there was a clear gradient of resistance with PIV5-CD55 being the most resistant, which was followed by PIV5-CD46 and then PIV5-CD59. Similar results were seen in dose-dependent neutralization assays with varying concentrations of NHS (see Figure 7B). This was most evident at the 1:20 and 1:40 dilutions of NHS where the resistance was in the order: PIV5-CD55, PIV5-CD46, PIV5-CD59, and PIV5-CHO. As shown in our prior study [33], CD55 was the most potent negative regulator, but there are differences between the potency shown in prior work and potency shown in this study due to donor-to-donor variation in neutralization capacity. These data indicated that PIV5-associated CD59 has less of an impact on complement-mediated neutralization than PIV5-associated CD46 or CD55.

### 3.5. PIV5 Infection Upregulates Cell Surface CD59 and Confers Virions with Increased Resistance to Complement-Mediated Neutralization

**Figure 8:**
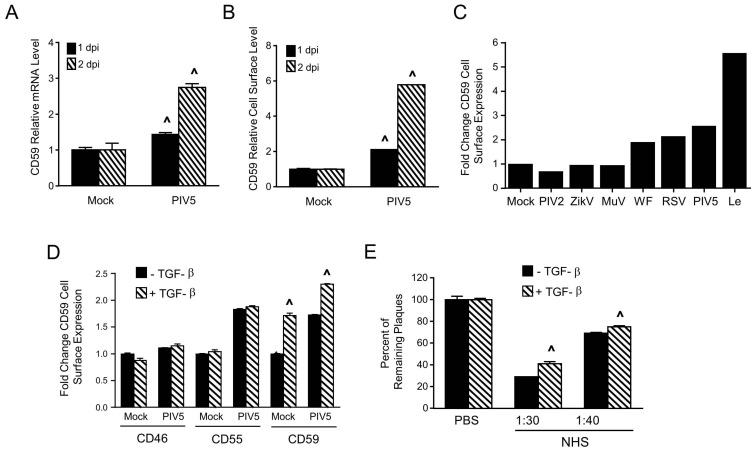
CD59 cell surface expression is upregulated following PIV5 infection, which results in viruses with higher resistance to complement-mediated neutralization. (**A**,**B**) A549 cells were mock infected or infected with PIV5 at an moi of 10. At 1 and 2 dpi, total RNA was isolated for qRT-PCR quantitation of relative levels of CD59 mRNA (panel (**A**)) or cells were analyzed for surface-expression of CD59 by flow cytometry (panel (**B**)). The data are from three independent reactions and error bars represent standard deviation. ˄ *p* < 0.01 when comparing PIV5-infected cells to mock infected cells. (**C**) A549 cells were mock infected or infected with the indicated viruses at an moi of 10 and levels of cell surface CD59 were quantitated by flow cytometry at 2 dpi. Results are an example of two independent experiments. (**D**) A549 cells were mock infected or infected with PIV5 at an moi of 10. At 16 hpi, cells were treated with 5 ng/mL of TGF-β1. At 2 dpi, levels of cell surface CD46, CD55, and CD59 were quantitated by flow cytometry. The data are from three independent reactions and error bars represent standard deviation. ˄ *p* < 0.01 when comparing TGF-treated cells to mock treated cells. (**E**) PIV5 was grown in untreated A549 cells or TGF-β1-treated cells. One hundred PFU of the resulting virus was incubated with PBS as a control or with the indicated dilutions of NHS for 1 h. Remaining infectivity was determined by using a plaque assay. The data are from three independent reactions and error bars represent standard deviation. ˄ *p* < 0.01 when comparing PIV5 derived from TGF-β1 treated cells to PIV5 derived from control cells.

We tested the hypothesis that PIV5 infection upregulates cell surface CD59 expression. To address this, A549 cells were mock infected or infected with PIV5 at an moi of 10. At 1 and 2 dpi, CD59 mRNA levels and cell surface CD59 expression were examined by qRT-PCR and flow cytometry, respectively. At 2 dpi, CD59 mRNA was induced by PIV5 infection ~3 fold compared to mock infected cells (see Figure 8A) and there was a ~6 fold increase in cell surface CD59 expression (see Figure 8B).

CD59 expression was induced by other RNA viruses. This is shown in Figure 8C. When A549 cells are infected with WT PIV5, the WF strain of PIV5 or the PIV5 leader mutant (Le) showed higher cell surface CD59 compared to the mock infection. A similar enhancement was seen with the RSV infection. By contrast, there was no increased CD59 expression following infection with PIV2, Zika virus, and MuV.

Proinflammatory cytokines such as tumor growth factor beta (TGF-β1) have been shown to induce cell surface CD59 expression [45]. To test if this also occurred in PIV5-infected cells, A549 cells were mock infected or infected with PIV5 at an moi of 10. At 16 hpi, cells were treated with 5 ng/mL of TGF-β1 for two days. Cell surface CD59 expression was examined by flow cytometry. As shown in Figure 8D, TGF-β1 treatment induced a statistically significant increase in cell surface CD59 expression in both mock and PIV5 infected A549 cells. Importantly, TGF-β1 treatment did not change levels of cell surface CD46 and CD55 expression.

PIV5 was grown in A549 cells with or without TGF-β1 treatment and the progeny virus harvested from infected cells was used for neutralization assays with varying concentrations of NHS. As shown in Figure 8E, PIV5 derived from TGF-β1-treated cells was more resistant to neutralization than PIV5 derived from untreated A549 cells at 1:30 and 1:40 dilutions of NHS. Given that CD46 and CD55 were not influenced by TGF-β1 treatment, our data are consistent with a proposal that PIV5 infection can upregulate CD59 expression at the cell surface, which results in progeny virions with increased resistance to complement-mediated neutralization.

## 4. Discussion

The complement system functions as the first line of innate immunity that rapidly responds to viral infection. Activation of the complement system by recognizing viruses leads to direct neutralization of infectivity and regulation of inflammatory and adaptive immune responses [1,2,3]. Therefore, understanding the mechanisms employed by viruses to inhibit complement pathways is a critical determinant of the outcome of viral neutralization as well as the potency of adaptive immune responses. Using a CHO cell line that stably expresses CD59, we show that PIV5 can incorporate an important host cell membrane-associated complement regulatory protein to limit complement-mediated neutralization. This suggests that incorporating CD59 into virions may be a common strategy for enveloped RNA viruses to limit complement pathways.

Our previous studies have shown that PIV5 can incorporate both CD46 and CD55 into progeny virions to inhibit complement-mediated neutralization [31,33]. In the case of MuV and VSV, CD55 is much more potent than CD46 in limiting complement-mediated neutralization [31,32], but the relative role of three important RCAs in PIV5 neutralization had not been addressed. In this study, we show that PIV5 can also associate with a functional CD59 to limit complement-mediated neutralization. Using cell lines that express only one RCA, we show that the potency of a given RCA to delay PIV5 neutralization followed a pattern of CD55 > CD46 > CD59.

We attempted to uncover why CD59 contributes little to a delay in PIV5 neutralization by complement. One possibility is that low levels of CD59 incorporation into progeny PIV5 relative to other RCAs contribute to lack of potency. This is supported by our finding that PIV5 derived from CHO-CD46 and CHO-CD55 cells contained ~0.03 μg of CD46/μg virus and ~0.1 μg of CD55/μg virus, respectively [33] while PIV5 derived from CHO-CD59 cells contained only ~0.014 ng of CD59/μg virus. Both CD55 and CD59 are attached to the membrane through glycophosphatidylinositol anchors, which raises the question of what determines virion incorporation of these two cell surface proteins. While no comparisons of relative surface expression to incorporate virions has been done, these data are consistent with low incorporation being a possible contributing factor to the low influence of CD59 delaying neutralization.

An alternative explanation for the low impact of CD59 on complement neutralization comes from the step in the complement cascade that is blocked by CD55, CD46, and CD59. For CD46 and CD55, inactivation of the complement pathway occurs at an upstream stage involving C3b and/or C4b while CD59 inhibits complement pathways at the terminal stage involving C5-C9 and formation of the MAC [13,46]. We have previously shown that, in most cases, PIV5 is neutralized predominantly by forming very large aggregates, which trap the virus in C3-rich complexes [36]. Consistent with this, we show that PIV5 can still be neutralized by C5 and C8 depleted sera. These data are consistent when interpreting that the low effect of CD59 on delaying complement-medicated neutralization of PIV5 is due to the small role for complement pathways downstream of C3.

PIV5 activates the complement system, but the interactions between complement and PIV5 infected cells are not fully understood. In this study, we show that PIV5-infected CHO cells activate complement pathways, which was evidenced by C3 being deposited on infected cells that are treated with NHS. In addition, we show that PIV5-infected CHO-CD59 cells have lower percentages of annexin V positive cells and less MAC deposition on infected cell surfaces when compared to PIV5-infected CHO control cells. These results have important implications for understanding the role of CD59 for the fate of PIV5 infected cells as well as viral tropism and dissemination.

Viruses such as human herpesvirus 7 have been shown to induce CD59 expression during infection of target cells [47]. In this study, we found that CD59 was upregulated by infection of A549 cells with PIV5-based viruses including PIV5 WT and the PIV5 Le-(U5C, A14G) mutant as well as by RSV infection. It is noteworthy that the PIV5 Le mutant contains alterations to the leader promoter, which lead to a much higher viral gene expression than WT PIV5 [35] and this higher expression of viral genes correlates with the highest induction of cell surface-associated CD59. These correlations suggest that specific viral gene products such as the viral glycoproteins or dsRNA generated during an infection might be strong inducers of CD59 pathways. Future work with the Le mutant will help elucidate the contributions of viral and cellular factors to CD59 induction.

Proinflammatory cytokines such as the tumor growth factor (TGF) has been shown to induce cell surface CD59 expression [45]. We have found that treatment of PIV5-infected A549 cells with TGF-β resulted in an increased level of cell surface CD59 expression over that of a virus infection alone. This observation raised the hypothesis that PIV5 derived from cells with increased cell surface CD59 expression would be more resistant to complement-mediated neutralization. PIV5 derived from TGF-β1 treated A549 cells was more resistant to neutralization than PIV5 derived from untreated A549 cells. It is noteworthy that TGF-β treatment had no effects on cell surface CD46 and CD55 expression in PIV5 infected cells, which led to the assumption that the small differences in neutralization between viruses from untreated and TGF-β treated cells was due to CD59. These results are similar to our prior work, which showed that treatment of A549 cells with exogenous TNF-alpha increased cell surface CD55, and virus from TNF-alpha treated cells had increased resistance to complement-mediated neutralization [33]. These results raise the interesting possibility that PIV5 takes advantage of the production of extracellular cytokines such as TGF and TNF to induce RCAs for increased resistance to complement. An interesting future direction is testing this hypothesis in an animal model and determining the role of cytokine-producing cells such as infiltrating lymphocytes in viruses acquiring resistance to complement.

## References

[1] Blue C.E., Spiller O.B., Blackbourn D.J. (2004). The relevance of complement to virus biology. Virology.

[2] Carroll M.C. (2004). The complement system in regulation of adaptive immunity. Nat. Immunol..

[3] Kemper C., Atkinson J.P. (2007). T-cell regulation: With complements from innate immunity. Nat. Rev. Immunol..

[4] Mehlhop E., Diamond M.S. (2006). Protective immune responses against West Nile virus are primed by distinct complement activation pathways. J. Exp. Med..

[5] Morrison T.E., Fraser R.J., Smith P.N., Mahalingam S., Heise M.T. (2007). Complement contributes to inflammatory tissue destruction in a mouse model of Ross River virus-induced disease. J. Virol..

[6] Delgado M.F., Polack F.P. (2004). Involvement of antibody, complement and cellular immunity in the pathogenesis of enhanced respiratory syncytial virus disease. Expert Rev. Vaccines.

[7] Stoermer K.A., Morrison T.E. (2011). Complement and viral pathogenesis. Virology.

[8] Johnson J.B., Aguilar H.C., Lee B., Parks G.D. (2011). Interactions of human complement with virus particles containing the Nipah virus glycoproteins. J. Virol..

[9] Bergmann-Leitner E.S., Leitner W.W., Tsokos G.C. (2006). Complement 3d: From molecular adjuvant to target of immune escape mechanisms. Clin. Immunol..

[10] Schauber-Plewa C., Simmons A., Tuerk M.J., Pacheco C.D., Veres G. (2005). Complement regulatory proteins are incorporated into lentiviral vectors and protect particles against complement inactivation. Gene Ther..

[11] Markiewski M.M., Lambris J.D. (2007). The role of complement in inflammatory diseases from behind the scenes into the spotlight. Am. J. Pathol..

[12] Roozendaal R., Carroll M.C. (2006). Emerging patterns in complement-mediated pathogen recognition. Cell.

[13] Kerr M.A. (1980). The human complement system: Assembly of the classical pathway C3 convertase. Biochem. J..

[14] Atkinson J.P., Liszewski M.K., Richards A., Kavanagh D., Moulton E.A. (2005). Hemolytic uremic syndrome: An example of insufficient complement regulation on self-tissue. Ann. N. Y. Acad. Sci..

[15] Pangburn M.K., Schreiber R.D., Muller-Eberhard H.J. (1983). Deficiency of an erythrocyte membrane protein with complement regulatory activity in paroxysmal nocturnal hemoglobinuria. Proc. Natl. Acad. Sci. USA.

[16] Kim D.D., Song W.C. (2006). Membrane complement regulatory proteins. Clin. Immunol..

[17] Meri S., Wldmann H., Lachmann P.J. (1991). Distribution of protectin (CD59), a complement membrane attack inhibitor, in normal human tissues. Lab. Investig..

[18] Shan C., Zhang S., Cui W., You X., Kong G., Du Y., Qiu L., Ye L., Zhang X. (2011). Hepatitis B virus X protein activates CD59 involving DNA binding and let-7i in protection of hepatoma and hepatic cells from complement attack. Carcinogenesis.

[19] Morgan B.P. (1999). Regulation of the complement membrane attack pathway. Crit. Rev. Immunol..

[20] Meri S., Morgan B.P., Davies A., Daniels R.H., Olavesen M.G., Waldmann H., Lachmann P.J. (1990). Human protectin (CD59), an 18,000–20,000 MW complement lysis restricting factor, inhibits C5b-8 catalysed insertion of C9 into lipid bilayers. Immunology.

[21] Rollins S.A., Sims P.J. (1990). The complement-inhibitory activity of CD59 resides in its capacity to block incorporation of C9 into membrane C5b-9. J. Immunol..

[22] Isaacs S.N., Kotwal G.J., Moss B. (1992). Vaccinia virus complement-control protein prevents antibody-dependent complement-enhanced neutralization of infectivity and contributes to virulence. Proc. Natl. Acad. Sci. USA.

[23] Chung K.M., Liszewski M.K., Nybakken G., Davis A.E., Townsend R.R., Fremont D.H., Atkinson J.P., Diamond M.S. (2006). West Nile virus nonstructural protein NS1 inhibits complement activation by binding the regulatory protein factor H. Proc. Natl. Acad. Sci. USA.

[24] Cummings K.L., Waggoner S.N., Tacke R., Hahn Y.S. (2007). Role of complement in immune regulation and its exploitation by virus. Viral Immunol..

[25] Saifuddin M., Hedayati T., Atkinson J.P., Holguin M.H., Parker C.J., Spear G.T. (1997). Human immunodeficiency virus type 1 incorporates both glycosyl phosphatidyinositor-anchored CD55 and CD59 and integral membrane CD46 at levels that protect from complement mediated destruction. J. Gen. Virol..

[26] Shaw M.L., Stone K.L., Colangelo C.M., Gulcicek E.E., Palese P. (2008). Cellular proteins in influenza virus particles. PLoS Pathog..

[27] Stoiber H., Pinter C., Siccardi A.G., Clivio A., Dierich M.P. (1996). Efficient destruction of human immunodeficiency virus in human serum by inhibiting the protective action of complement factor H and decay accelerating factor (DAF, CD55). J. Exp. Med..

[28] Vanderplasschen A., Mathew E., Hollinshead M., Sim R.B., Smith G.L. (1998). Extracellular enveloped vaccinia virus is resistant to complement because of incorporation of host complement control proteins into its envelope. Proc. Natl. Acad. Sci. USA.

[29] Hirsch R.L., Wolinsky J.S., Winkelstein J.A. (1986). Activation of the alternative complement pathway by mumps infected cells: Relationship to viral neuraminidase activity. Arch. Virol..

[30] McSharry J.J., Pickering R.J., Caliguiri L.A. (1981). Activation of the alternative complement pathway by enveloped viruses containing limited amounts of sialic acid. Virology.

[31] Johnson J.B., Grant K., Parks G.D. (2009). The paramyxoviruses simian virus 5 and mumps virus recruit host cell CD46 to evade complement-mediated neutralization. J. Virol..

[32] Johnson J.B., Lyles D.S., Alexander-Miller M.A., Parks G.D. (2012). Virion-associated CD55 is more potent than CD46 in mediating resistance of mumps virus and VSV to neutralization. J. Virol..

[33] Li Y., Johnson J.B., Parks G.D. (2016). Parainfluenza virus 5 upregulates CD55 expression to produce virions with enhanced resistance to complement-mediated neutralization. Virology.

[34] Young V.A., Dillon P.J., Parks G.D. (2006). Variants of the paramyxovirus Simian virus 5 with accelerated or delayed viral gene expression activate proinflammatory cytokine synthesis. Virology.

[35] Manuse M.J., Parks G.D. (2009). Role for the paramyxovirus genomic promoter in limiting host cell antiviral responses and cell killing. J. Virol..

[36] Johnson J.B., Capraro G.A., Parks G.D. (2008). Differential mechanisms of complement mediated neutralization of the closely related paramyxoviruses simian virus 5 and mumps virus. Virology.

[37] Li Y., Kakinami C., Li Q., Yang B., Li H. (2013). Human apolipoprotein A-I is associated with dengue virus and enhances virus infection through SR-BI. PLoS ONE.

[38] Conde J.N., da Silva E.M., Allonso D., Coelho D.R., Andrade I.D., de Medeiros L.N., Menezes J.L., Barbosa A.S., Mohana-Borges R. (2016). Inhibition of the Membrane Attack Complex by Dengue Virus NS1 through Interaction with Vitronectin and Terminal Complement Proteins. J. Virol..

[39] Zhang Z., Yang J., Wei J., Yang Y., Chen X., Zhao X., Gu Y., Cui S., Zhu X. (2011). Trichinella spiralis paramyosin binds to C8 and C9 and protects the tissue-dwelling nematode from being attacked by host complement. PLoS Negl. Trop. Dis..

[40] Brasoveanu L.I., Altomonte M., Fonsatti E., Colizzi F., Coral S., Nicotra M.R., Cattarossi I., Cattelan A., Natali P.G., Maio M. (1996). Levels of cell membrane CD59 regulate the extent of complement-mediated lysis of human melanoma cells. Lab. Investig..

[41] Jarvis G.A., Li J., Hakulinen J., Brady K.A., Nordling S., Dahita R., Meri S. (1997). Expression and function of the complement membrane attack complex inhibitor protectin (CD59) in human prostate cancer. Int. J. Cancer.

[42] Chen S., Caragine T., Cheung N.K., Tomlinson S. (2000). CD59 expressed on a tumor cell surface modulates decay-accelerating factor expression and enhances tumor growth in a rat model of human neuroblastoma. Cancer Res..

[43] Coral S., Fonsatti E., Sigalotti L., De Nardo C., Visintin A., Nardi G., Colizzi F., Colombo M.P., Romano G., Altomonte M. (2000). Overexpression of protectin (CD59) down-modulates the susceptibility of human melanoma cells to homologous complement. J. Cell. Physiol..

[44] Jurianz K., Ziegler S., Garcia-Schüler H., Kraus S., Bohana-Kashtan O., Fishelson Z., Kirschfink M. (1999). Complement resistance of tumor cells: Basal and induced mechanisms. Mol. Immunol..

[45] Goswami M.T., Reka A.K., Kurapati H., Kaza V., Chen J., Standiford T.J., Keshamouni V.G. (2016). Regulation of complement-dependent cytotoxicity by TGF-β-induced epithelial–mesenchymal transition. Oncogene.

[46] Song W.C. (2004). Membrane complement regulatory proteins in autoimmune and inflammatory tissue injury. Curr. Dir. Autoimmun..

[47] Takemoto M., Yamanishi K., Mori Y. (2007). Human herpesvirus 7 infection increases the expression levels of CD46 and CD59 in target cells. J. Gen. Virol..

